# Transitioning from a First Generation to Second Generation Large-Scale Agile Development Method: Towards Understanding Implications for Coordination

**DOI:** 10.1007/978-3-030-58858-8_9

**Published:** 2020-08-18

**Authors:** Finn Olav Bjørnson, Torgeir Dingsøyr

**Affiliations:** 6 IT University of Copenhagen, Copenhagen, Denmark; 7 University of British Columbia, Vancouver, BC Canada; 8 Norwegian University of Science and Technology, Trondheim, Norway; 9 SINTEF Digital, Trondheim, Norway

**Keywords:** Large-scale agile, Coordination

## Abstract

This paper reports our initial findings from a longitudinal case study within a large development project in a public organization in Scandinavia. We focus on changes in coordination practices as the development project moved from a 1^st^ to a 2^nd^ generation large-scale agile development methodology. Building on four theories of coordination from different fields, we investigate how each theory illuminates our case and what insight they might provide. We find that two of the theories are well suited to characterizing each phase, providing answer to *how* coordination was done. While two other theories can provide answers to *why* these changes occurred.

## Introduction

Large-scale agile software development has received significant interest in the last years [1, 2]. In particular, the topic of how to coordinate many development teams has been seen as critical to the success of agile development at scale. A previous study identified coordination challenges in large-scale agile development due to misaligned planning at inter-team levels [3]. The introduction to the special issue on large-scale agile development in *IEEE Software,* describes two generations of large-scale agile development methods:

*The first generation* of large-scale agile development methods combined agile methods at team level with traditional project management frameworks such as PRINCE2. *A second generation* of large-scale agile development methods are currently taken up globally. These methods which include Disciplined Agile Delivery, Large-Scale Scrum, the Scaled Agile Framework and the Spotify model [2] prescribe new arenas as well as roles in order to ensure coordination.

Agile methods have significant impact on coordination practices. They “*de*-*emphasize traditional coordination mechanisms such as forward planning, extensive documentation, specific coordination roles, contracts, and strict adherence to a pre*-*defined specified process*” [4]. In a previous article, we argued that coordination has to be re-thought [5] in order to emphasize a number of characteristics of large-scale agile development including focus on oral communication, work in teams, a high level of interdependencies, uncertainty in tasks, many people involved, relations between individuals and that coordination needs change over time.

Today, many organisations and projects are transitioning from a first to a second-generation method for large-scale agile development. In this paper, we discuss the following research question: *How can theories on coordination explain changes in coordination when moving from a first*- *to a second generation large*-*scale agile development method?*

We draw on a longitudinal case study of a development project in a large public organization in a Scandinavian country. The project started with a first generation large-scale agile method. However, in the last phase of the project, they changed to a second-generation model with autonomous teams. In the following, we first present theory which has previously been used to study coordination in large-scale agile development, then our case study method, some initial results from the case study and then a discussion of around using identified theory in analysing this case. We conclude with what we see as preliminary recommendations for studying transitions of coordination practices in large-scale agile development.

## Theory

A previous article [5] identified four theories we believe are relevant in order to develop research-based advice for the software industry on coordination. In Table 1 below, we briefly present each theory with key reference and with reference to studies using this theory on large-scale agile development. Note that the theories have very different origins. The theory from Strode [4] and from Salas et al. [6] have basis in single teams, while Van de ven [7] and Jarzabkowski [8] focuses on organisations.

**Table 1.** Four coordination theories, adapted from [5]

Field	Description	References
Software Engineering (Strode et al.)	A coordination strategy consists of three components: Synchronization: In arenas such as daily meetings where team members meet at the same time and place. Structure: Physical closeness, team member availability and that team members can substitute others. Boundary spanning: Activities, artefacts and roles to coordinate with other people or units beyond the project	Key reference: [4]
Sociology (van de Ven et al.)	Coordination is done through persons (“personal mode”) or through artefacts (“impersonal mode”). If coordination is done through persons, it could be done individually or in groups. Impersonal coordination is “programmed” or “codified” for example through plans or written coding standards	Key reference: [7] Used in: [9–11]
Organizational Psychology (Salas et al.)	Mechanisms for coordination on team level are seen as relevant for inter-team coordination in multiteam systems. Three coordination mechanisms: Shared mental models: Common understanding of tasks, work process and knowledge of others. Closed-loop communication: Senders of messages ensure that messages are received correctly. Mutual trust: Shared belief that team members will perform roles and protect interests of teammates	Key reference: [6] Used in: [12–14]
Management Science (Jarzabkowski et al.)	Management Science researchers refer to the process of Coordinating to underscore the dynamic and emergent characteristics of coordination mechanisms. Jarzabkowski et al. argue that coordinating mechanisms are subject for change, are established, fall apart, and are transformed over time	Key reference: [8]

## Method

Our data is based on a longitudinal case study of a development project in PublicOrg. In order to explain the setting, we first provide an overview of PublicOrg and the development project, before commenting briefly on our research method for understanding the case.

### Case

The fieldwork was conducted within a development project at PublicOrg, a large Scandinavian organization for public services. The IT department develops, operates, and manages IT solutions that support close to 20,000 employees in their work, and provide solutions for about 800,000 active users. The IT department has approximately 700 employees and 400 consultants and maintains and operates close to 300 applications.

One of the core systems was originally developed in 1978. To accommodate legislative changes and support increased automation of work, the organization began a series of modernization projects in 2012. The projects would replace the old system in three increments. The first of these projects failed, and the failure resulted in massive media attention. When the second project began in 2016, stakeholders were therefore determined to avoid failure at any cost. This project, the “Beta” project, which is the one we are studying, had an estimated cost of 130 million Euro. At the start of our fieldwork, PublicOrg’s strategy for IT development was to outsource software development projects to external suppliers. The suppliers, usually consultancy companies, would then be responsible for development and maintenance, while PublicOrg was responsible for coordination and operations. The IT department at PublicOrg employed a staged development method, with formal handovers between stages.

Our unit of analysis is the Beta project, the project was organized and run by PublicOrg according to their traditional model. They created a project group and hired one consultancy company to help them with formulating requirements and another consultancy company to implement the solution. At the height of the project group including the consultancy companies involved approximately 200 people.

Since the previous project had been a failure, much attention was given to control mechanisms and to ensure accountability within this second project. At the same time, the IT-department at PublicOrg hired a new leader who had new ideas of how PublicOrg should manage their IT strategy. They would move away from the previous regime of outsourcing and more towards inhouse development, taking ownership of their own systems. However, while the head of PublicOrg IT was talking about lean business driven development in autonomous teams in an environment of trust and openness, the project was still underway under the old regime of control and formal handovers. This change in strategy by PublicOrg IT would affect the Beta project gradually.

### Research Method

The study of the Beta project has been organized as a longitudinal case study. Several researchers have been involved with gathering data over a period of two years. Our material consists of observations, project documents, and over 30 semi-structured interviews usually lasting from 30 min to two hours. The interviews were recorded and transcribed. Our main source of informants were the people from the consultancy company hired to develop the solution, but we also conducted interviews with key people in PublicOrg and in the consultancy company hired to assist with the requirements.

We are currently in the process of organizing and coding the material. The findings in this paper is based on our initial understanding of the case and the material before beginning a thorough analysis. Our analysis will consist of combination of bottom up and top down coding, and this paper outlines some key theories and constructs which will be used in the top down part of the analysis.

## Results

During the *first phase* of the development project, which was based on the first generation “Perform model” [15, 16], there were several structures designed to promote coordination at the inter team level. We have identified around 20 arenas and tools used during this phase. Formal meetings like the upstart meetings in the beginning of each iteration, and Scrum of Scrums intended to keep the teams in the loop on what was happening in other teams. Architectural forums and test forums sought to keep the specialized roles in each team in touch with each other. Artefacts such as the overall architecture and dependency map contributed to handling of dependencies between teams. In addition, many team members were rotated between teams as the project was scaling up in order to promote knowledge sharing between developers. Contact between the developers and PublicOrg was largely formal, each team had a “functional architect” from their consultancy company that was the contact person towards the product owners and solution architects at PublicOrg. The product owners and solution architects were situated in another part of the building. We label this first part of the project “First generation agile development methodology”, since the agility of the development teams takes place in strict boundaries within a framework of project management and waterfall methodology.

For the *second phase* of the project not much was changed for the core development teams. They were scaling up eventually ending up with seven teams. There were some changes relating to how they communicated with PublicOrg, as the product owners and solution architects were moved physically closer to the teams they were working with, but handover of user stories was still much based on a “relay race” approach. The larger change for this phase was that a part of the system was spun off from the larger solution and given to a dedicated multidisciplinary team, “Team Bravo”. This was in addition to the seven core teams. Team Bravo was not locked into the reporting, testing and deployment regime of the core teams, they had autonomy to deploy to production at will. This team was not part of any of the coordination arenas the core teams were using to coordinate their dependencies, and dependencies with the core system was handled on an ad hoc basis, leading to some frustration as expressed by a project manager: *“We were not quite in phase when it came to coordination, I*’*d say. And I bet they thought we were daft, and we thought they were a bit daft too. But such things pass easy, given some time”*. This second phase of the project we’ve labeled “Bimodal”, since the core teams are continuing with their original methodology, while team Bravo is trying out a new way of working.

During the second phase of the project, the decision was made to change the entire organization of the project, this happened in the *third phase* Everybody should now work in multidisciplinary teams. A change that was characterized as *“Changing the engine of an airplane in flight with 200 passengers aboard”*. Every team had autonomy to decide their own work structure, and most of the coordination arenas across teams was dropped in addition to several middle management roles being removed. PublicOrg hired two agile coaches that would advise the teams on how they could work. From two major deployments a year, they would now move to daily deployments. The teams were structured around functional areas of the overall system so every team had a dedicated area to develop and support. We characterize this third phase of the development project as a “second generation agile development model” as in this phase the teams have a much larger degree of both agility and responsibility.

## Discussion

We return to our initial research question: *How can theories on coordination explain changes in coordination when moving from a first generation to a second generation large*-*scale agile development method?*

From the theoretical perspective of Strode [4] we see that the synchronization has been left to the teams in the new structure. Very little emphasis has been on synchronization structures across teams and six months after the change, informants were expressing needs for more synchronization arenas across teams. Structure has been well kept in that all members of teams are sitting together and they are physically close to the other teams. However, team member availability across teams can be a challenge, as can teams’ ability to substitute each other since every team is now specialized within an application area. Boundary spanning is where we can see a clear lack of structure in the new organization, which might have implications later. However, six months after phase three began, we saw new boundary spanning mechanisms emerging to meet this need.

Looking at the case with the theoretical perspective of van de Ven [7], we see a move from group mode of personal coordination towards individual mode of personal coordination. In addition, we see a move within group mode coordination from scheduled to unscheduled meetings. Since decisions are moved towards the team level, we see a decline in impersonal modes of coordination across teams since teams are no longer bound by guidelines developed by other teams.

The theoretical lens of Salas [6] allows us to explain why we continue to see good coordination between teams despite removing many arenas of cross team coordination. The main explanation lies in the development of a shared mental model during the first phases of the project. With this in mind, developers are able to coordinate across teams because they know what the other teams are doing. It will be interesting to see if the new structure will lead to a decline in the shared mental model over time, which some informants were indicating signs of six months after the transition to phase three. With the teams being given much autonomy, trust is probably the second most influential factor. The management trusts the teams and the teams trust each other. The shared mental model and trust might also be key constructs in explaining the poor coordination between the first autonomous team and the rest of the developing teams during the second phase of the project.

Finally, the theoretical perspective of Jarzabkowski [8] allows us to understand the process of rebuilding the coordination arenas after they were removed during the change in the project organization. After the reorganization most of the focus was on establishing coordination at the intra team level, but when we visited the project six months after the transition, multiple respondents expressed interest in re-establishing some of the coordination mechanisms at the inter team level.

It seems like these four theories align themselves along two major lines of questions for this case. Strode and van de Ven are useful to characterize the phases and answer questions relating to *how* coordination practices were organized during the project. The theories of Salas and Jarzabkowski are useful to describe *why* these changes occurred, and the apparent success of the transition.

The main limitation of this paper is that we are presenting initial findings before the main analysis is done. We do, however, believe the findings are important and interesting in that they are guiding our work on structuring the analysis, and what types of questions each theory might provide answers to. Other researchers may find the constructs from the identified theories useful in guiding their own research towards coordination in large-scale development methods. Practitioners may find it useful to use the constructs of Strode and van de Ven to map their own current practices and keep the constructs of Salas and Jarzabkowski in mind when designing a transition strategy.

## Conclusion and Further Work

In this paper we have reported on some of our initial findings relating to coordination from a large scale development project that transitioned from a first to a second generation development methodology during the project.

We used four different theoretical perspectives, originally put forward in [5], to analyse our findings. We found that two theories [4, 7] were useful for characterizing the phases of the transition and answer questions relating to *how* coordination was done. The other two theories [6, 8] were useful to describe *why* the changes occurred, and could help us explain the apparent success of the transition. From our initial findings, a well developed shared mental model and sufficient degree of trust seems to be key factors in the successful coordination while transitioning from one generation to the next.

From a research point of view, the identified concepts might be useful in informing new studies of coordination in large-scale software development. From a practitioner point of view we offer guidance on how to categorize the current strategy and offer some key concepts to keep in mind when designing a transition strategy.

Moving forward, we will continue to analyse the material from our longitudinal study more in depth from the different theoretical perspectives we have identified, we might also broaden our study with the theory of Relational Coordination, recently suggested in [17] or specifically focus on coordination artefacts [18]. Our aim is to both provide input to practitioners looking for evidence-based advice on scaling their agile methods, as well as continuing our discourse on rethinking coordination in large-scale software development.

## References

[1] Bass JM, Hoda Rashina (2019). Future trends in agile at scale: a summary of the 7th international workshop on large-scale agile development. Agile Processes in Software Engineering and Extreme Programming – Workshops.

[2] Dingsøyr T, Falessi D, Power K (2019). Agile development at scale: the next frontier. IEEE Softw..

[3] Bick S, Spohrer K, Hoda R, Scheerer A, Heinzl A (2018). Coordination challenges in large-scale software development: a case study of planning misalignment in hybrid settings. IEEE Trans. Softw. Eng..

[4] Strode DE, Huff SL, Hope BG, Link S (2012). Coordination in co-located agile software development projects. J. Syst. Softw..

[5] Dingsøyr, T., Bjørnson, F.O., Moe, N. B., Rolland, K., Seim, E.A.: Rethinking coordination in large-scale software development, Gothenburg, Sweden, pp. 91–92 (2018). 10.1145/3195836.3195850

[6] Salas E, Sims DE, Burke SC (2005). Is there a “Big five” in teamwork?. Small Group Res..

[7] Van de Ven AH, Delbecq AL, Koenig R (1976). Determinants of coordination modes within organizations. Am. Sociol. Rev..

[8] Jarzabkowski PA, Le JK, Feldman MS (2012). Toward a theory of coordinating: creating coordinating mechanisms in practice. Organ. Sci..

[9] Moe NB, Dingsøyr T, Rolland K (2018). To schedule or not to schedule? An investigation of meetings as an inter-team coordination mechanism in large-scale agile software development. Int. J. Inf. Syst. Proj. Manage..

[10] Dingsøyr T, Moe NB, Seim EA (2018). Coordinating knowledge work in multi-team programs: findings from a large-scale agile development program. Proj. Manage. J..

[11] Nyrud, H., Stray, V.: Inter-team coordination mechanisms in large-scale agile. In: Proceedings of the XP2017 Scientific Workshops, pp. 1–6 (2017)

[12] Bjørnson, F.O., Wijnmaalen, J., Stettina, C. J., Dingsøyr, T.: Inter-team coordination in large-scale agile development: a case study of three enabling mechanisms. In: XP2018, Porto, Portugal, pp. 216–231 (2018)

[13] Scheerer, A., Hildenbrand, T., Kude, T.: Coordination in large-scale agile software development: a multiteam systems perspective. In: 2014 47th Hawaii International Conference on System Sciences, pp. 4780–4788 (2014)

[14] Scheerer, A., Kude, T.: Exploring coordination in large-scale agile software development: a multiteam systems perspective. In: Proceedings of the International Conference on Information Systems (2014)

[15] Dingsøyr T (2019). Key lessons from tailoring agile methods for large-scale software development. IEEE IT Prof..

[16] Dingsøyr T, Moe NB, Fægri TE, Seim EA (2017). Exploring software development at the very large-scale: a revelatory case study and research agenda for agile method adaptation. Empir. Softw. Eng..

[17] Berntzen M, Moe NB, Stray V, Kruchten P, Fraser S, Coallier F (2019). The product owner in large-scale agile: an empirical study through the lens of relational coordination theory. Agile Processes in Software Engineering and Extreme Programming.

[18] Zaitsev A, Gal U, Tan B (2020). Coordination artifacts in agile software development. Inf. Organ..

